# Biostimulant and antagonistic potential of endophytic fungi against fusarium wilt pathogen of tomato *Fusarium oxysporum* f. sp*. lycopersici*

**DOI:** 10.1038/s41598-024-66101-1

**Published:** 2024-07-04

**Authors:** Marie Cecile Muhorakeye, Everlyne Samita Namikoye, Fathiya M. Khamis, Waceke Wanjohi, Komivi S. Akutse

**Affiliations:** 1 International Centre of Insect Physiology and Ecology (icipe), P.O. Box 30772-00100, Nairobi, Kenya; 2 Department of Agricultural Science and Technology, Kenyatta University, P.O. Box 43844-00100, Nairobi, Kenya; 3 Rwanda Polytechnic, Integrated Polytechnic Regional College (IPRC) Musanze, P.O. Box 226, Musanze, Rwanda; 4 Unit of Environmental Sciences and Management, North-West University, Private Bag X6001, Potchefstroom, 2520 South Africa

**Keywords:** Fusarium wilt, Endophyte, Phytopathogen suppression, Growth inhibition, Defence genes expression, Biological techniques, Ecology, Microbiology, Plant sciences

## Abstract

Endophytic fungal-based biopesticides are sustainable and ecologically-friendly biocontrol agents of several pests and diseases. However, their potential in managing tomato fusarium wilt disease (FWD) remains unexploited. This study therefore evaluated effectiveness of nine fungal isolates against tomato fusarium wilt pathogen, *Fusarium oxysporum* f. sp*. lycopersici* (FOL) in vitro using dual culture and co-culture assays. The efficacy of three potent endophytes that inhibited the pathogen in vitro was assessed against FWD incidence, severity, and ability to enhance growth and yield of tomatoes *in planta.* The ability of endophytically-colonized tomato (*Solanum lycopersicum* L.) plants to systemically defend themselves upon exposure to FOL were also assessed through defence genes expression using qPCR. In vitro assays showed that endophytes inhibited and suppressed FOL mycelial growth better than entomopathogenic fungi (EPF). Endophytes *Trichoderma asperellum* M2RT4, *Hypocrea lixii* F3ST1, *Trichoderma harzianum* KF2R41, and *Trichoderma atroviride* ICIPE 710 had the highest (68.84–99.61%) suppression and FOL radial growth inhibition rates compared to EPF which exhibited lowest (27.05–40.63%) inhibition rates. Endophytes *T. asperellum* M2RT4, *H. lixii* F3ST1 and *T. harzianum* KF2R41 colonized all tomato plant parts. During the *in planta* experiment, endophytically-colonized and FOL-infected tomato plants showed significant reduction of FWD incidence and severity compared to non-inoculated plants. In addition, these endophytes contributed to improved growth promotion parameters and yield. Moreover, there was significantly higher expression of tomato defence genes in *T. asperellum* M2RT4 colonized than in un-inoculated tomato plants. These findings demonstrated that *H. lixii* F3ST1 and *T. asperellum* M2RT4 are effective biocontrol agents against FWD and could sustainably mitigate tomato yield losses associated with fusarium wilt.

## Introduction

Globally, the agriculture industry is endangered by numerous plant pests and diseases, and their prevalence is increasing^1^. Farmers spend more than 40% of their crop production cost on pest control, while up to 50–82% reduction in attainable yield in the major crop has been attributed to both pests and diseases^2–4^. Tomato (*Solanum lycopersicum* L.), being the second most cultivated vegetable worldwide is among threatened commodities^5^. For instance, in Kenya, tomato production plays a vital role in improving lives, particularly for smallholder farmers, through employment and household income generation^6,7^. The crop accounts for approximately 14% and 6.72% of Kenya’s total vegetable and horticultural production, respectively^8^. In terms of local market sales, tomato makes up approximately 20% of total vegetable production in Kenya and contributes nearly 158.4 million US dollars annually to the Kenyan economy^9^. The potential yield of this crop in Kenya can reach up to 30.7 t ha^−1^^10^. However, pests, diseases, and physiological disorders reduce the crop's achievable productivity by up to 12 tonnes per hectare^8,11^. According to Singh et al.^12^ and Ma et al.^13^, tomato is prone to approximately more than 200 diseases of which soil-borne diseases are the most prevalent.

Fusarium wilt is the most frequent soil-borne disease hindering tomato production^13^. It could cause up to 10–80% loss in the total production of tomatoes thereby threatening the livelihood of small-scale farmers^8,13^. The pathogenic fungus *Fusarium oxysporum* f. sp. *lycopersici*, which causes this disease enters the host roots and invades its vascular tissues, preventing water and nutrient uptake^14,15^. This results in wilting of one side of the plant, starting with the older leaves and progressing to the death of the entire host plant^15,16^. Moreover, the brown vascular darkening discolouration in cross sections of the stem tissue and stem decay are observed, especially in wet conditions^17^. Furthermore, the pathogen produces resting structures “chlamydospores” that can survive in soil for a decade, making its control extremely difficult^15^.

The management options of fusarium wilt range from cultural, resistant, chemical, and botanical to biological control^16^. Initially, uprooting infected plants and the use of resistant varieties was the safe, effective, and affordable strategy for farmers^16^. However, uprooting is laborious and finding resistant cultivars is difficult owing to the breakdown of resistance due to the various races of this pathogen^16,18^. Soil solarization and application of chemical pesticides such as methyl bromide, benomyl, or carbendazim are used to control the pathogen. However, significant concerns about their harmful effects on humans, animals, the environment and biodiversity have been raised^11,19^. To address these challenges, eco-friendly and sustainable strategies such as biological control that can effectively manage fusarium wilt in tomatoes are paramount^13,20^.

Biological control involves the use of living organisms (biocontrol agents) such as entomopathogenic fungi (EPF) and endophyte-based biopesticides in the sustainable management of crop pests and diseases^21–23^. These biological control agents (BCAs) like endophytes, grow faster and occupy the root zone of the plant before the pathogenic fungi are established thereby promoting plant health, triggering plant defence system and resistance to pathogen attack^13,24–26^. Recently, several EPF and endophytes such as *Metarhizium anisopliae*, *Beauveria bassiana*, *Trichoderma* spp. isolates and *Hypocrea lixii* have been shown to endophytically colonize tomato plants, promote growth, and protect the host plants against pests and pathogens^27,28^. Furthermore, yield improvement and systemic resistance to fusarium wilt were reported in tomato plants endophytically colonized by BCAs *Trichoderma asperellum* and *B. bassiana*^26^.

In addition, these BCAs especially endophytes benefit the host plants by activating their defence genes in endophytically colonized tomato plants and this was evidenced by higher expression levels of pathogenesis-related proteins and enzymes in comparison to untreated tomato plants^24,26,29,30^. For instance, the increased level of expression of pathogenesis-related proteins- PR-1 was seen in FOL-infected tomato plants pre-treated with *T. asperellum* strains TS-12 and TS-39^24^. Induction of defence-related *SWRKY* gene transcripts was found in tomato plants endophytically colonized with *Trichoderma erinaceum* and infected with FOL^31^. Tomato lipoxygenase (*TomLoxC*), which is responsible for jasmonic acid (JA) biosynthesis, was found to be highly expressed in tomato plants pre-treated with *Trichoderma* isolates and inoculated with *Pseudomonas syringae* pv. *Tomato*, and *Botrytis cinerea,* respectively^32,33^. Furthermore Prasetyawan et al.^34^ and Ueki et al.^35^, reported the role of pathogenesis-related proteins-PR-2 (β-1,3-glucanase) in suppressing a variety of fusarium wilt pathogens. Although several *Trichoderma* species' defence mechanisms have been studied, there is a gap in the proper understanding of this topic, as the effectiveness of these BCAs/endophytes varies from species to species, from one strain to another within a species, and is influenced by environmental factors^36,37^.

Recent studies reported for the antagonistic effects of fungal isolates from various genera such as *Bacillus*, *Streptomyces*, *Trichoderma*, *Hypocrea*, *Beauveria*, and *Metarhizium* against plants pests and pathogens^13,26,38–43^. However, knowledge of the effectiveness of locally isolated EPF and endophytes to sustainably manage fusarium wilt is largely unknown. Additionally, the mechanisms used by these microbial biocontrol agents to stimulate tomato plant growth and yield and confer resistance against fusarium wilt have not been explored. Hence, this study aimed to contribute to the sustainable management of fusarium wilt disease of tomato crops in Kenya using entomopathogenic fungi and endophyte-based biopesticides. Consequently, an in vitro assessment of some selected entomopathogenic and endophytic fungal isolates against *Fusarium oxysporum lycopersici* fungal pathogen was conducted. In addition, the efficacy of the potent fungal isolates on the occurrence and severity of fusarium wilt disease in tomato crops under greenhouse conditions was evaluated. Furthermore, the study assessed the expression levels of some selected defence genes in tomato plants endophytically colonized by *T. asperellum* M2RT4 when exposed to *Fusarium oxysporum lycopersici.*

## Results

### Identification of Fusarium wilt pathogen for bioassays

The isolated fungus was identified as *Fusarium oxysporum* f. sp. *lycopersici* (FOL) based on key morphological features described by Leslie and Summerell^44^, such as white colony color and purplish-pink pigmentation (Fig. S1A and B). Three types of spores—microconidia, macroconidia, and chlamydospores—were observed under a microscope (40× magnification). The pathogen was successfully re-isolated from inoculated tomato roots and stems, with 100% and 80% colonization rates, respectively. Besides, the re-isolated pathogen exhibited similar morphological features to the inoculated FOL mother plates following slide preparation for comparison/confirmation test. Furthermore, using ITS 4 and ITS 5, EF1 α, and RPB1 primers, the molecular testing confirmed the identification of the pathogen as FOL with 100%, 99.23%, and 98.64% nucleotide similarity to the FOL sequences deposited to GenBank of accession numbers MT530269.1, XM_018381269.1, and XM_018377523.1, respectively.

### Antagonistic effect of the selected fungal biocontrol agents against *Fusarium oxysporum lycopersici*

**Figure 1:**
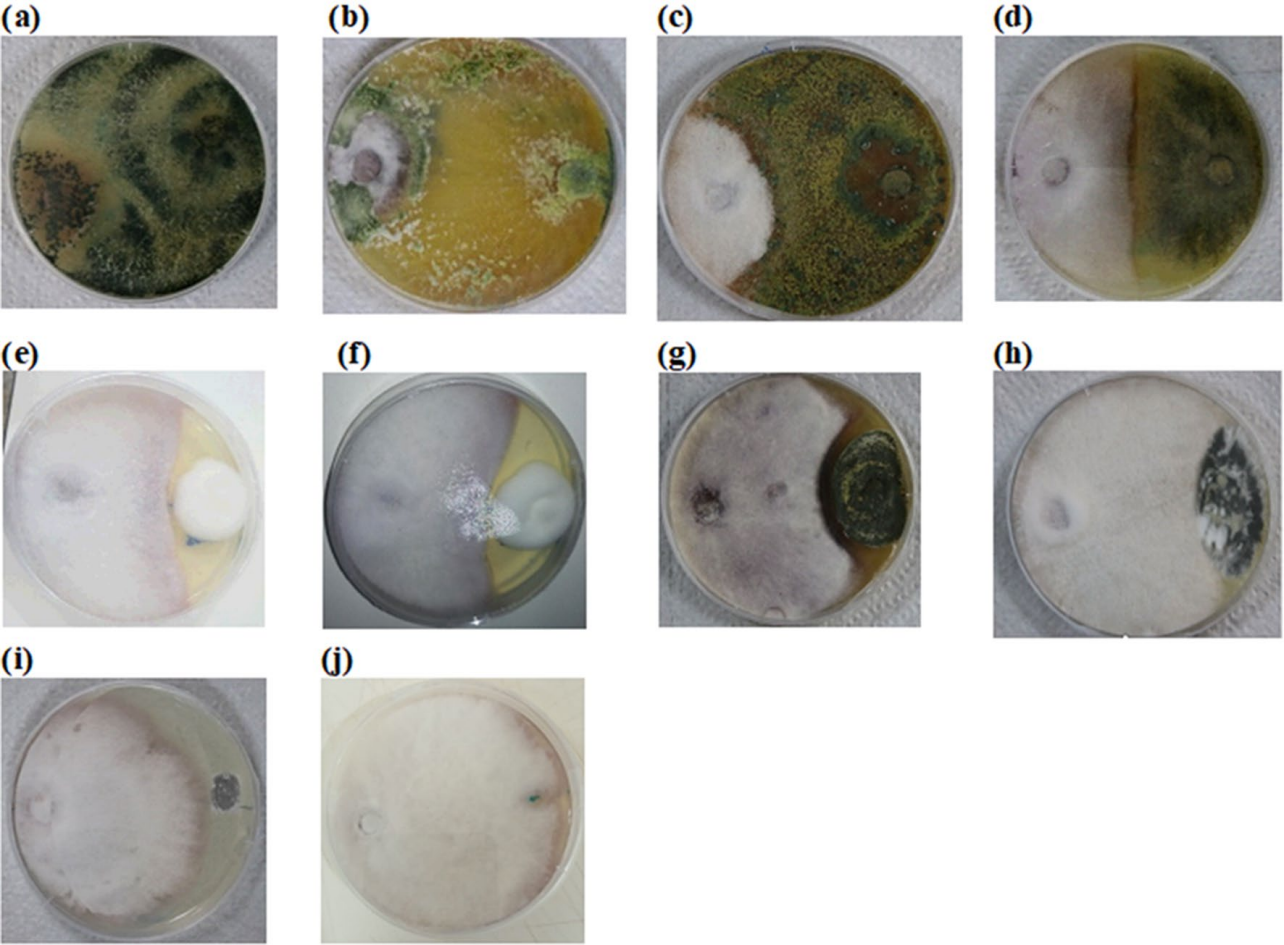
Inhibitory and suppressive effects of selected biocontrol agents against *Fusarium oxysporum lycopersici* mycelia growth on the 12th day of their dual culture assay: (**a**) *Trichoderma asperellum* M2RT4, (**b**) *Hypocrea lixii* F3ST1, (**c**) *Trichoderma harzianum* KF2R41, (**d**) *Trichoderma atroviride* ICIPE 710, and (**e**) *Beauveria bassiana* ICIPE 706, (**f**) *Beauveria bassiana* ICIPE 273, (**g**) *Metarhizium anisopliae* ICIPE 20, (**h**) Control, (**i**) *Metarhizium anisopliae* ICIPE 18, (**j**) *Metarhizium anisopliae* ICIPE 665.

**Figure 2:**
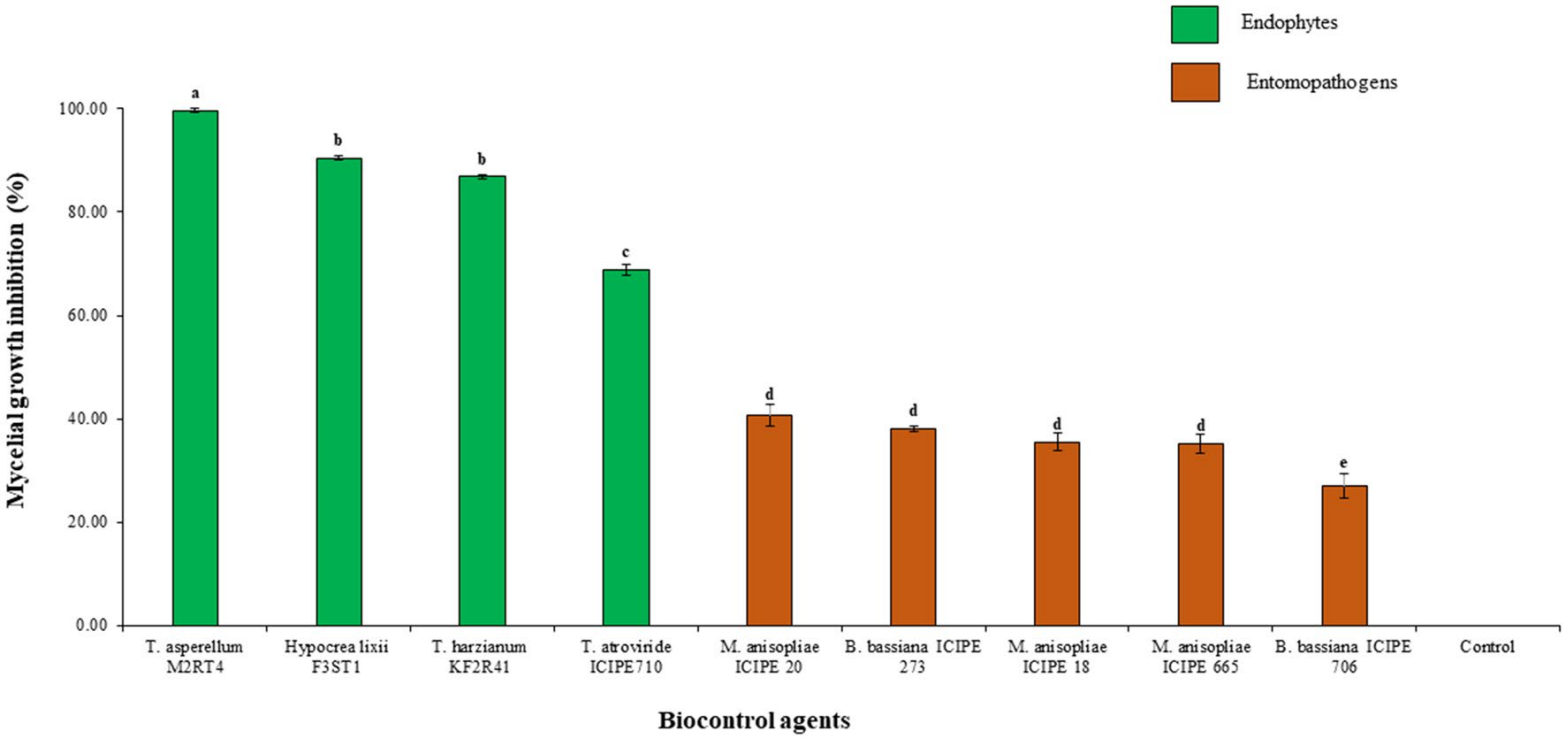
Average percentage (mean ± SE) of mycelial growth inhibition of four endophytic and five entomopathogenic fungal isolates against *Fusarium oxysporum lycopersici* on the 12th day of their dual culture assay*.* Different small letters above the error bars denote significant differences across the treatment means.

The endophytic fungal isolates showed high potential in inhibiting and suppressing the pathogen compared to EPF (Fig. 1). A significant difference (χ^2^ = 2277.7, df = 8, *P* < 0.001) was observed among the nine screened fungal BCAs against FOL (Fig. 2). Of all the four endophytic fungal isolates, *T. asperellum* M2RT4 had the highest inhibition and suppression of radial growth of FOL, while *T. atroviride* ICIPE 710 had the lowest. *Trichoderma asperellum* M2RT4, *H. lixii* F3ST1, *T. harzianum* KF2R41, and *T. atroviride* ICIPE 710 achieved 99.61%, 90.5%, 86.84%, and 68.84% FOL mycelia growth inhibition, respectively. Among EPF, *M. anisopliae* ICIPE 20 had the highest inhibition rate (40.63%) of FOL followed by *B. bassiana* ICIPE 273 (38.10%), *M. anisopliae* ICIPE 18 (35.53%), *M. anisopliae* ICIPE 655 (35.23%), while the lowest inhibition rate (27.05%) of FOL mycelia growth was obtained in the dual culture of FOL and *B. bassiana* ICIPE 706 (Fig. 2).

### Co-culture of fungal biocontrol agents and test pathogen

All the endophytic fungal isolates were dominant in terms of mycelia and pigments produced in their co-culture with FOL on the 7th day post-inoculation. In contrast, FOL mycelia and pigment predominated on co-cultured EPF and FOL plates. Among all fungal BCAs, only *T. asperellum* M2RT4 suppressed/inhibited the growth and expression of the mycelia of FOL in their co-cultured plates on day 14 after incubation. Similarly, dominance in mycelial morphology was observed in co-cultures of *T. harzianum* KF2R41, *H. lixii* F3ST1, and *T. atroviride* ICIPE 710 with FOL; however, a small amount of FOL mycelia were also observed in these co-cultures. On the other hand, FOL mycelia and its pigmentation were entirely dominant in its co-culture with *M. anisopliae* isolates ICIPE 18, ICIPE 20, and ICIPE 655, although mycelia were more abundant in the co-culture of FOL and *M. anisopliae* ICIPE 665. Furthermore, white spores of *B. bassiana* were observed in the co-cultured plates of *B. bassiana* isolates ICIPE 273 and ICIPE 706 and FOL; however, FOL mycelia and pigmentation were dominant (Fig. S2).

### Endophytic colonization assessment

**Figure 3:**
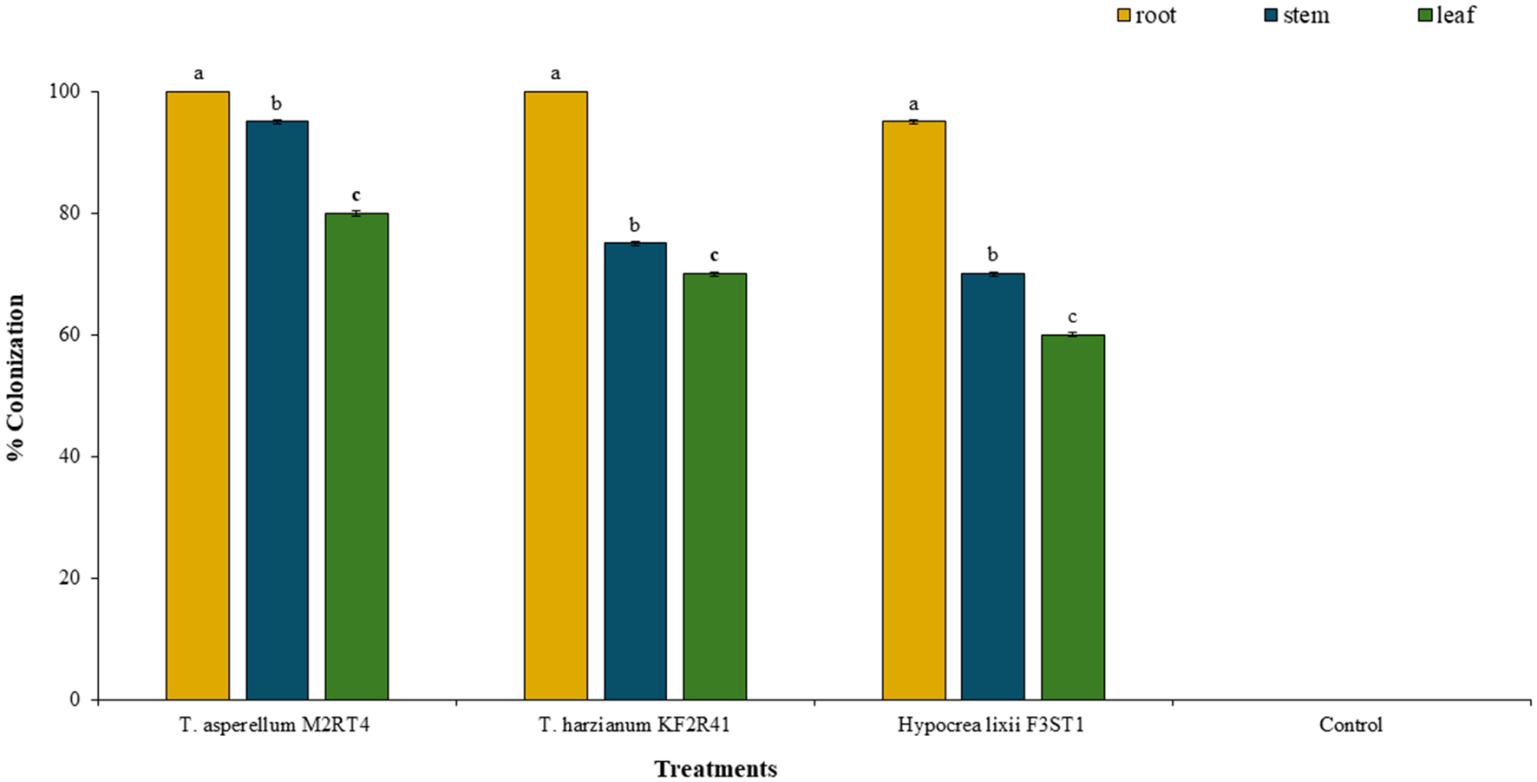
Mean % colonization of the different tomato (*Solanum lycopersicum* L.) plant parts by the three selected potent endophytic fungal isolates.

Before the *in planta* bioassay, the endophytic colonization of the identified potent endophytes which suppressed the pathogen in vitro was assessed or confirmed through seed inoculation. Significant differences in the tomato seedling colonization by the various endophytic isolates were observed on roots (χ^2^ = 6, df = 2, *P* = 0.04979), stems (χ^2^ = 26.571, df = 2, *P* < 0.001), and leaves (χ^2^ = 33.273, df = 2, *P* < 0.001) (Fig. 3). *Trichoderma asperellum* M2RT4, *T. harzianum* KF2R41, and *H. lixii* F3ST1 colonized all the tomato plant parts and were effectively re-isolated from inoculated tomato seedlings. However, the rate of colonization varied among isolates and plant sections/tissues. Among the isolates, *T. asperellum* M2RT4 demonstrated high potential of colonizing all tomato plant parts at high rates; however, the roots exhibited the highest rate of colonization rate (100%) compared to stems (90%) and leaves (80%) (Fig. 3). A similar trend was seen in the colonization rate of *T. harzianum* KF2R41, where the colonization rates of roots, stems, and leaves were 100%, 75%, and 70%, respectively. Furthermore, *H. lixii* F3ST1 colonized tomato roots, stems, and leaves at the rates of 95, 70, and 60%, respectively (Fig. 3).

### Effect of the selected potent fungal endophytes on fusarium wilt disease incidence and severity *in* *planta*

**Figure 4:**
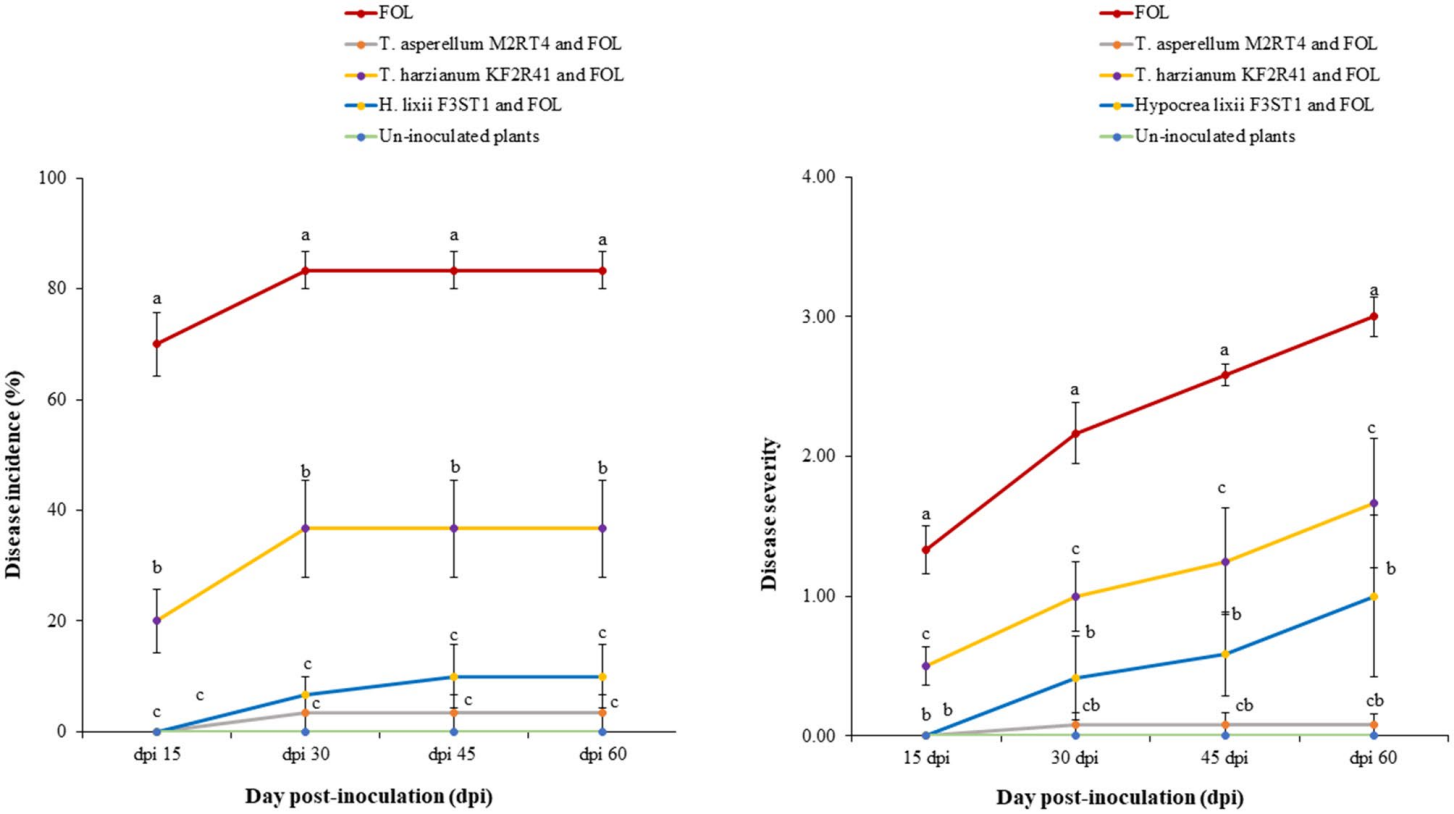
Effects of selected potent fungal endophytes *in planta* on fusarium wilt disease. Mean percentage and score of fusarium wilt incidence and severity at 15-, 30-, 45- and 60-days post-inoculation (dpi)**. (A)** disease incidence, (**B)** disease severity. Each value represents the mean ± SE. Means followed by different letters are significantly different across the treatments at 95% CI (SNK test, *P* ≤ 0.05, n = 8).

Tomato plants inoculated with endophytes *T. asperellum* M2RT4, *H. lixii F3ST1*, and *T. harzianum* KF2R41 significantly-reduced the incidence and severity of fusarium wilt disease at 15-, 30-, 45- and 60-days post-inoculation (dpi) compared to the FOL-infected plants (*P* < 0.001). Of all the treatments, *T. asperellum* M2RT4 proved to be the most effective with the lowest disease incidence (1.66%) and severity (0.04/4) (Fig. 4A and B). The highest incidence (80%) and severity (2.7/4) of fusarium wilt disease were recorded in FOL-inoculated plants. The continuous increase in disease incidence up to 30 dpi and disease severity up to 60 dpi were also recorded in FOL-inoculated plants (Tables 1 and 2). Only moderate symptoms and decreased fusarium disease incidence (6.6%, 32.5%) and severity (0.5/4, 1.1/4) were observed in *H. lixii* F3ST1 and *T. harzianum* KF2R41 inoculated plants, respectively. The greatest Area Under Disease Progress Curve (AUPDC) was obtained in tomato plants infected with FOL without pre-treatment with endophytes. This was followed by FOL-infected tomato plants pretreated with endophytes *T. harzianum* KF2R41, *H. lixii* F3ST1, *T. asperellum* M2RT4, showing AUPDC values of 1250, 562.5, and 78.125, respectively (Table 3). Furthermore, there was no incidence nor disease severity recorded in non-inoculated plants (Fig. 4A and B).

**Table 1:** Fusarium wilt disease incidence reduction in tomato plants pre-treated with the selected potent endophytes.

Treatments	Disease incidence at dpi 60 (%)	Disease incidence reduction (%)
FOL	83.33	–
*T. asperellum* M2RT4 and FOL	3.33	96
*T. harzianum* KF2R41 and FOL	36.67	56
*H. lixii* F3ST1 and FOL	10	88
Un-inoculated plants	0	–

**Table 2:** Fusarium wilt disease severity reduction in tomato plants pre-treated with the selected potent endophytes.

Treatments	Disease severity at dpi 60 (%)	Disease severity reduction (%)
FOL	3.00	–
*T. asperellum* M2RT4 and FOL	0.08	97.33
*T. harzianum* KF2R41 and FOL	1.67	44.44
*H. lixii* F3ST1 and FOL	1.00	66.67
Un-inoculated plants	0.00	–

**Table 3:** Aggressiveness of *Fusarium oxysporum lycopersici* based on disease severity (%) and Area Under Disease Progress Curve (AUDPC) value on tomato plants pretreated with the selected endophytes at different day post inoculation (dpi).

	Disease severity (%)				AUDPC			
	1st evaluation	2nd evaluation	3rd evaluation	4th evaluation				
Treatments	15	30	45	60	15–30 dpi	30–45 dpi	45–60 dpi	Total AUDPC
Non-inoculated	0.00	0.00	0.00	0.00	0.00	0.00	0.00	0.00
FOL	33.33	54.17	64.58	75.00	656.25	890.63	1046.88	2593.75
*T. asperellum* M2RT4 and FOL	0.00	2.08	2.08	2.08	15.63	31.25	31.25	78.13
*T. harzianum* KF2R41 and FOL	12.50	25.00	31.25	41.67	281.25	421.88	546.88	1250.00
*H. lixii* F3ST1 and FOL	0.00	10.42	14.58	25.00	78.13	187.50	296.88	562.50

### Growth enhancement in endophytically-colonized and FOL-infected tomato plants

**Figure 5:**
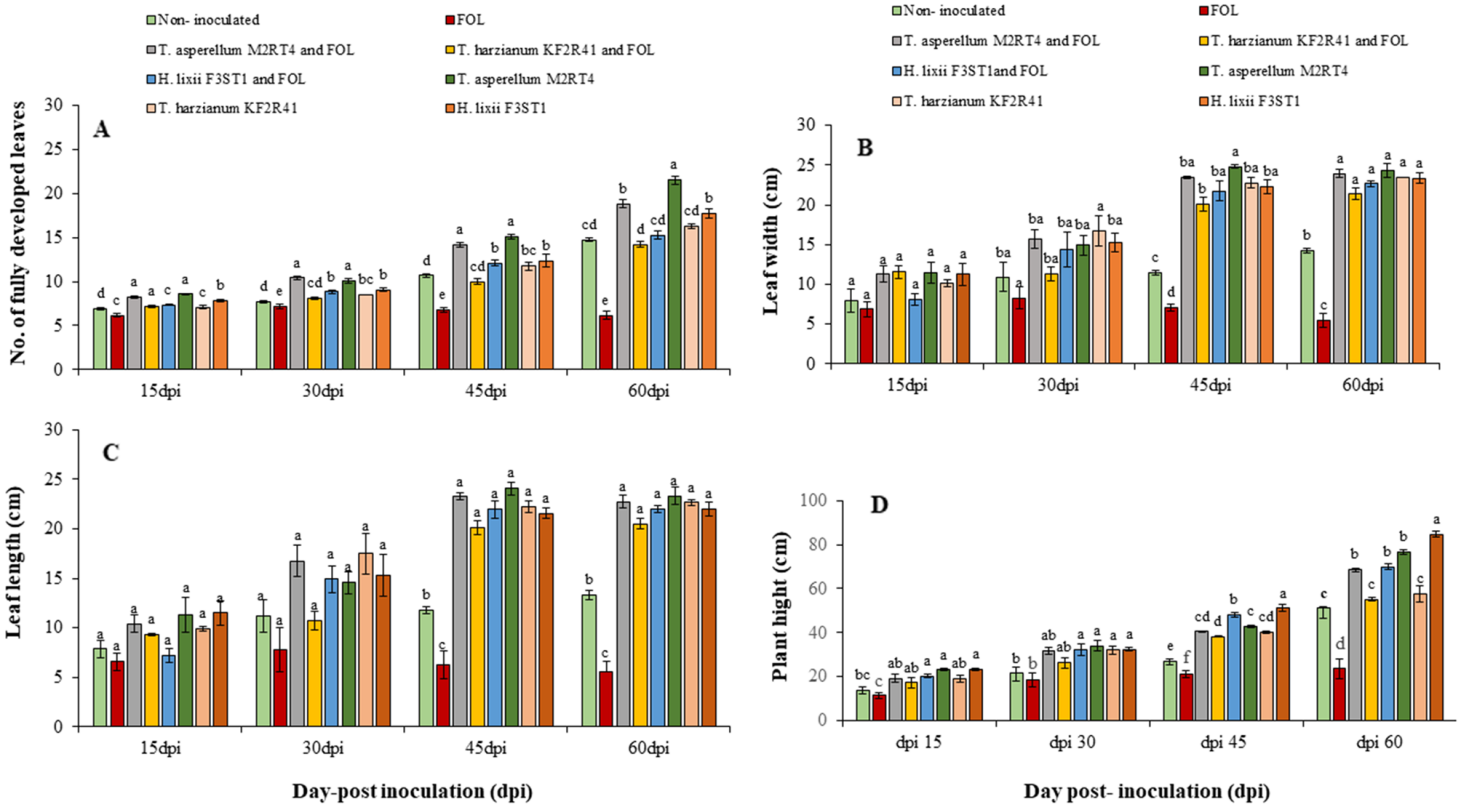
Effect of the selected potent fungal endophytes on the growth of FOL-infected tomato plants at 15-, 30-, 45- and 60-days post-inoculation (dpi). (**A**) Number of fully developed leaves, (**B**) Leaf width, (**C**) Leaf length, (**D**) Plant height. The bars represent mean ± standard error. Means followed by different small letters are significant differences across the treatment at 95% CI (SNK test, *P* ≤ 0.05, n = 8).

There were significant differences in all evaluated growth parameters of tomato plants pre-treated with the selected potent endophytes, infected with FOL, endophytically-colonized and FOL- infected, and un-inoculated tomato plants across all the assessment periods: 15-, 30-, 45- and 60-days post-inoculation (dpi) (Fig. 5). Significant differences in the number of fully developed leaves were observed among the treatments at 15, 30, 45, and 60 dpi (χ^2^ = 221.09, df = 7, *P* < 0.001; χ^2^ = 238.26, df = 7, *P* < 0.001; χ^2^ = 320.55, df = 7, *P* < 0.001; χ^2^ = 802.98, df = 7, *P* < 0.001), respectively (Fig. 5A). The highest number of fully developed leaves (18.83 ± 0.51) was recorded in *T. asperellum* M2RT4 endophytically-colonized tomato plants, while FOL-infected tomato plants had the lowest (6.16 ± 0.42). Furthermore, from 45 to 60 dpi, the number of fully developed leaves decreased from 6.75 to 6.16 (± 0.42) in FOL-infected plants. Time series analysis revealed significant differences (χ^2^ = 1780.391, df = 7, *P* < 0.001; χ^2^ = 3522.2, df = 3, *P* < 0.01) in number of fully developed leaves across treatments and all sampling periods, respectively.

The leaf width differed significantly in response to the various treatments across all the post-inoculation periods (15, 30, 45 and 60 dpi) as the tomato plants were growing (χ^2^ = 22.361, df = 7, *P* < 0.001; χ^2^ = 25.463, df = 7, *P* < 0.001; χ^2^ = 589.11, df = 7, *P* < 0.001; χ^2^ = 834.65, df = 7, *P* < 0.001), respectively (Fig. 5B). The result of time series analysis showed significant differences in leaf width and all the post-inoculation periods (χ^2^ = 563.1, df = 7,* P* < 0.001; χ^2^ = 603.59, df = 3, *P* < 0.001), respectively. A similar trend was observed in leaf length in the endophytically-colonized, FOL-infected, endophytically-colonized and FOL-infected, and un-inoculated tomato plants at 15, 30, 45, 60 dpi (χ^2^ = 26.1, df = 7, *P* < 0.001; χ^2^ = 27.079, df = 7, *P* < 0.001; χ^2^ = 508.91, df = 7, *P* < 0.001; χ^2^ = 635.65, df = 7, *P* < 0.001), respectively (Fig. 5C). However, the leaf length did not differ at 15 and 30 dpi. Besides, significant differences (χ^2^ = 760.61, df = 7,* P* < 0.001; χ^2^ = 2404.78, df = 3, *P* < 0.001) in leaf length and all sampling periods were observed among the treatments, respectively.

Significant differences (χ^2^ = 52.809, df = 7, *P* < 0.001; χ^2^ = 43.178, df = 7, *P* < 0.001; χ^2^ = 1206.4, df = 7, *P* < 0.001; χ^2^ = 480.45, df = 7, *P* < 0.001) in plant height were observed among treatments at 15, 30, 45 and 60 dpi, respectively (Fig. 5D). The highest plant height (84.75 ± 1.38 cm) was recorded in *H. lixii* F3ST1-colonized tomato plants, while the lowest was recorded in FOL-infected plants (23.66 ± 2.57 cm). There were also significant differences in plant heights of endophytically-colonized, FOL-infected, endophytically colonized and FOL-infected, as well as un-inoculated tomato plants across all sampling periods (χ^2^ = 760.61, df = 7,* P* < 0.001; χ^2^ = 2404.78, df = 3, *P* < 0.001), respectively (Fig. 5D). Furthermore, the interaction of treatments and sampling periods was significantly different (*P* < 0.001) in all evaluated tomato growth parameters.

The results of plant biomass accumulation are provided in Table 4. There was a significant difference (χ^2^ = 603.54, df = 7,* P* < 0.001) in the dry weight of the roots of endophytically-colonized, FOL-infected, endophytically-colonized, FOL-infected, and un-inoculated tomato plants. Similarly, a significant difference (χ^2^ = 780.72, df = 7, *P* < 0.001) was observed in the dry weight of shoots across various treatments. The dry weight of the whole plant also differed significantly (χ^2^ = 179.01, df = 7,* P* < 0.001) among the treatments. Overall, tomato plants pre-treated with endophytes displayed an increased dry weight of roots and shoots. However, the dry weight of the whole plant was higher in *H. lixii* F3ST1 and *T. asperellum* M2RT4 pre-treated plants.

**Table 4:** Mean dry weight of root, shoot and the whole plant (g) of endophytically-colonized and *Fusarium oxysporum lycopersici* (FOL)-infected tomato plants.

Treatments	Mean dry weight of root (g)	Mean dry weight of shoot (g)	Mean dry weight of plant (g)
No-inoculated tomato plants	1.73 ± 0.04^c^	6.82 ± 0.09^d^	8.56 ± 0.13^e^
FOL	1.40 ± 0.02^c^	6.02 ± 0.44^d^	7.42 ± 0.44^e^
*T. asperellum* M2RT4 and FOL	2.99 ± 0.36^ba^	13.17 ± 0.38^b^	16.16 ± 0.72^cb^
*T. harzianum* KF2R41 and FOL	1.80 ± 0.04^c^	11.12 ± 0.45^c^	12.93 ± 0.42^d^
*H. lixii* F3ST1 and FOL	2.93 ± 0.16^a^	12.87 ± 0.16^b^	15.80 ± 0.13^c^
*T. asperellum* M2RT4	3.52 ± 0.52^a^	14.86 ± 0.33^a^	18.64 ± 0.64^cb^
*T. harzianum* KF2R41	2.03 ± 0.15^b^	11.92 ± 0.13^cb^	13.96 ± 0.09^a^
*H. lixii* F3ST1	3.34 ± 0.31^a^	15.11 ± 0.36^a^	18.20 ± 0.54^ba^
*P value*	< 0.001	< 0.001	< 0.001

Mean ± standard error followed by different letters within a column indicate significant differences between the treatments at the *P* < 0.05 (n = 8) by SNK test.

### Effects of selected endophytes on FOL-infected tomato plants yield

The results of the effects of endophytes on the yield performance of the various treatments of tomato plants are shown in Table 5. There were significant differences in the number of fruits per plant, individual fruit weight, and yield (T ha^−1^) of endophytically colonized, FOL-infected, endophytically-colonized and FOL-infected, and un-inoculated tomato plants (χ^2^ = 173.42, df = 7, *P* < 0.001; χ^2^ = 550.87, df = 7, *P* < 0.001; χ^2^ = 619.88, df = 7, *P* < 0.001), respectively. Number of fruits per plant, individual fruit weight and yields were increased by 38.10%, 33.94%, and 59.02%, respectively, in FOL-infected tomato plants pre-treated with endophyte *T*. *asperellum* M2RT4 compared to control plants (Table 6). A similar trend was also observed in the evaluated yield parameters of FOL-infected tomato plants pre-treated with endophyte *T. harzianum* KF2R41, where percent increase in the number of fruits plants per plant, individual fruit weight, and yield were 25.19%, 20.09%, and 40.17%, respectively (Table 6). Furthermore, a percentage increase number of fruits per plant (26.77%), fruit weight (43.74%), and yield (58.74%) were recorded in FOL-infected tomato plants pre-treated with *H. lixii* F3ST1 (Table 6). On the other hand, *Fusarium oxysporum lycopersici* had a negative impact on all of the examined yield metrics in infected tomato plants by lowering the number of fruits per plant, individual fruit weight, and yield.

**Table 5:** Means of the tomato fruit attributes after application of potent endophytic fungal isolates. FOL: *Fusarium oxysporum lycopersici.*

Treatments	Mean no. of fruit per plant	Mean single fruit weight (g)	Mean yield (kg/plant)	Mean yield (T/ha)
Non-inoculated	20.78 ± 0.74^c^	38.92 ± 1.23^c^	0.81 ± 0.01^c^	32.29 ± 0.5^c^
FOL	17.33 ± 0.51^d^	29.77 ± 0.69^d^	0.52 ± 0.02^d^	20.65 ± 0.95^d^
*T. asperellum* M2RT4 and FOL	28.00 ± 1.00^a^	45.06 ± 0.85^b^	1.26 ± 0.02^a^	50.40 ± 0.9^a^
*T. harzianum* KF2R41 and FOL	23.16 ± 0.86b	37.25 ± 1.34^c^	0.86 ± 0.05^c^	34.52 ± 1.85^c^
*H. lixii* F3ST1 and FOL	23.66 ± 0.87^b^	52.91 ± 0.57^a^	1.25 ± 0.04^a^	50.06 ± 1.51^a^
*T. asperellum* M2RT4	28.99 ± 0.51^a^	45.00 ± 0.75^b^	1.30 ± 0.01^a^	52.17 ± 0.38^a^
*T. harzianum* KF2R41	24.33 ± 0.32^b^	44.06 ± 1.07^b^	1.07 ± 0.04^b^	42.90 ± 1.56^b^
*H.* lixii F3ST1	23.99 ± 0.88^b^	54.46 ± 0.43^a^	1.31 ± 0.04^a^	52.25 ± 1.43^a^
*P value*	< 0.001	< 0.001	< 0.001	< 0.001

Mean ± standard error followed by different letters within a column indicate significant differences between the treatments at the *P* < 0.05 (n = 8) by SNK test.

**Table 6:** The percentage yield increase in *Fusarium oxysporum lycopersici* (FOL)-infected tomato plants pre-treated with the selected potent fungal endophytes.

Treatments	Mean no. of fruits per plant	% increase in the of fruit	Mean individual fruit weight	% increase in individual fruit weight	Mean yield T/ha	% increase in yield (T/ha)
Non-inoculated	20.78	–	38.92	–	32.29	–
FOL	17.33	–	29.77	–	20.65	–
*T. asperellum* M2RT4 and FOL	28.00	38.10	45.06	33.94	50.40	59.02
*T. harzianum* KF2R41 and FOL	23.17	25.19	37.25	20.09	34.52	40.17
*H. lixii* F3ST1 and FOL	23.67	26.77	52.91	43.74	50.06	58.74

In general, tomato plants endophytically colonised by *T. asperellum* M2RT4 recorded the highest number of fruits, while the highest fruit weights were obtained in *H. lixii* F3ST1 colonized tomato plants. There were no significant differences in mean yield between *T. asperellum* M2RT4 and *H. lixii* F3ST1 endophytically-colonized and FOL-infected tomato plants and their positive controls in all the evaluated yield parameters. Furthermore, tomato yield did not differ considerably when *T. harzianum* KF2R41 endophytically-colonized and FOL-infected tomato plants were compared to non-inoculated tomato plants.

### Defence gene expression in endophytically-colonized tomato plants and or plants infected by *Fusarium oxysporum lycopersici*

**Figure 6:**
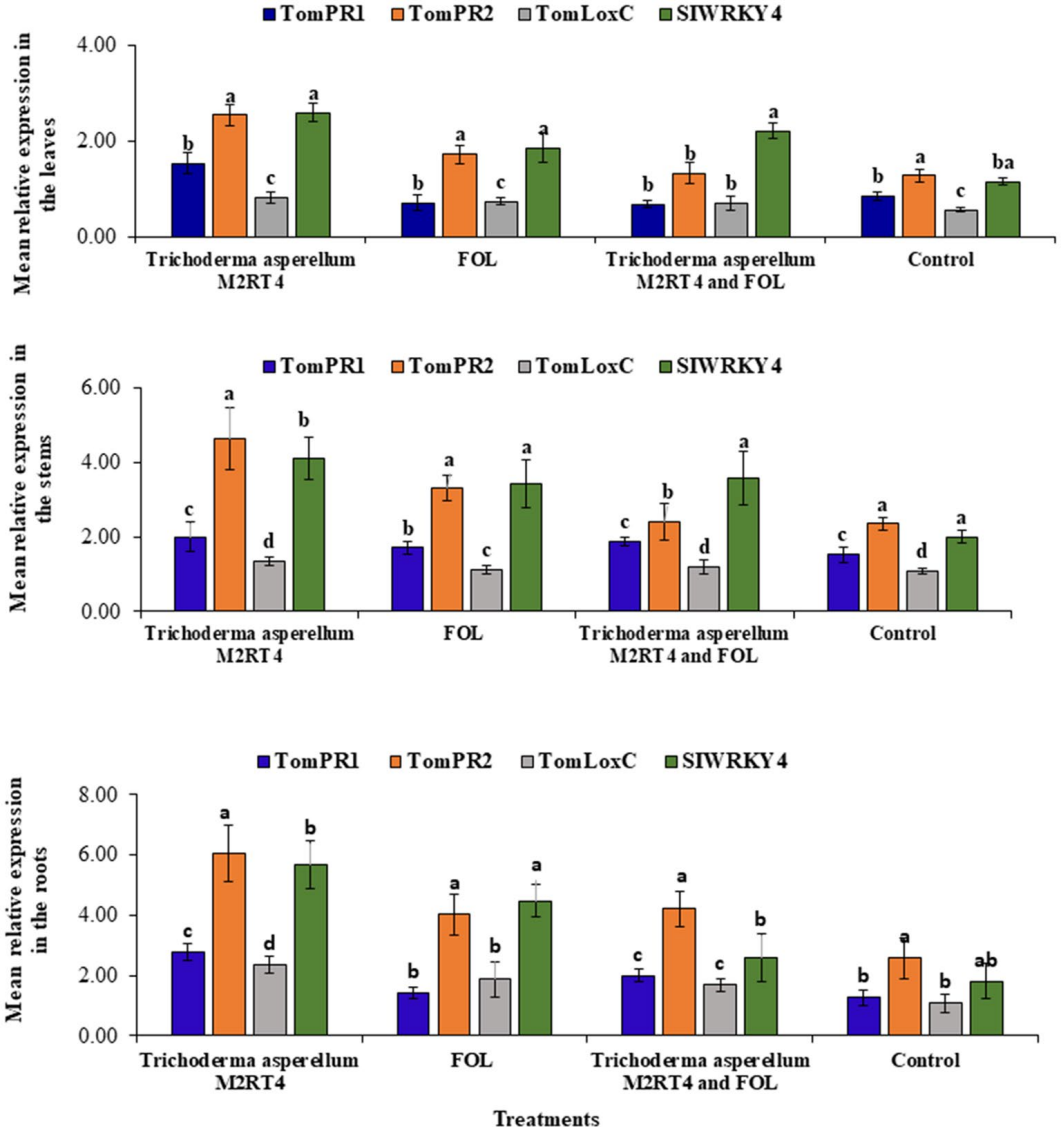
Relative gene expression of four specific genes associated with tomato plant defence mechanisms using the 2^−ΔΔCT^ method. The expression was observed in the leaves (**A**), stems (**B**) and roots (**C**) treatments.

The expression level of the four selected tomato defence genes: TomPR1, TomPR2, TomLoxC, and SIWRKY4 in roots, stems and leaves of *T. asperellum* M2RT4 endophytically colonized, FOL-infected, *T. asperellum* M2RT4 endophytically-colonized and FOL- infected, as well as un-inoculated tomato plants are presented in (Fig. 6). Tomato GAPDH was used to normalise the gene expressions utilising the 2^−ΔΔCT^ method^45^ and the selected genes did not have influence in the leaf treatments (df = 3, *P* = 0.3404), the stem treatments (df = 3, *P* = 0.1198) and the root treatments (df = 3, *P* = 0.08529). Consequently, there was an observed upregulation of the 4 selected defence genes (pathogenesis-related genes (TomPR1 and TomPR2), Loxigenase C (TomLoxC) and SWIRKY4) in the leaves (Fig. 6A), with the mean relative gene expression of SIWRKY4 gene (2.60 ± 0.20) being highest from the *T. asperellum* M2RT4 treatment, followed by TomPR2 gene (2.55 ± 0.23), TomPR1 (1.54 ± 0.22) and the least observed in TomLoxC gene (0.82 ± 0.12) in the control treatment.

The upregulation of the assessed defence genes was also observed in stems of *T. asperellum* M2RT4 endophytically colonized, FOL-infected, *T. asperellum* M2RT4 endophytically-colonized and FOL- infected, as well as un-inoculated tomato plants (Fig. 6B), with the mean relative gene expression of TomPR2 gene (4.65 ± 0.83) being highest from the *Trichoderma asperellum* M2RT4 treatment, followed by SIWRKY4 gene (4.10 ± 0.57) and the least observed in TomLoxC gene (1.08 ± 0.07) in the control treatment. In terms of treatments, the mean relative gene expression of the *T. asperellum* M2RT4 treatment was most elevated in the TomPR2 gene (4.65 ± 0.83) and SIWRKY4 gene (4.10 ± 0.57) compared to 2.35 ± 0.17 and 2.00 ± 0.17, respectively in the control treatment. In all four treatments, the mean relative gene expression of TomPR2 and SIWRKY4 seemed to be equal while was least in the TomLoxC gene (Fig. 6B). However, the upregulation observed was not statistically significant across all four treatments (df = 9, *P* = 1.0000).

A similar trend was observed in the root treatments (Fig. 6C) whereby the mean relative gene expression in the *Trichoderma asperellum* M2RT4 treatment was highest in TomPR2 gene (6.05 ± 0.95) followed by SIWRKY4 gene (5.69 ± 0.80) with least expression observed in TomLoxC gene (2.35 ± 0.29) as compared to 2.58 ± 0.68, 1.80 ± 0.58 and 1.08 ± 0.31, respectively from the control treatment. Although there was upregulation of the four selected genes in the roots, the variances were not statistically significant (df = 9, *P* = 0.99988) across all the treatments.

## Discussion

The use of entomopathogenic (EPF) and endophytic fungi (EF) as a safe and effective biocontrol strategy to combat plant pests and diseases while enhancing crop performance has gained global attention^13,16,26^. Nevertheless, knowledge of the effectiveness of locally isolated EPF and EF to sustainably manage fusarium wilt in tomato plants is largely unknown^16,41^. Additionally, the mechanisms used by these biological control agents (BCAs) to improve tomato growth and yield while conferring resistance against fusarium wilt pathogen are not well explored^37^. In this study, the causal agent of fusarium wilt of tomato was isolated, identified and used as a test pathogen to assess the antagonistic effects of nine BCAs in *Metarhizium*, *Beauveria*, *Hypocrea* and *Trichoderma* genera under in vitro, as well as their *in planta* ability to control this disease and enhance tomato growth and yield. Furthermore, the mechanism used by *T. asperellum* M2RT4, the most potent isolate, to overcome the negative effects of FOL on infected tomato plants was evaluated using q-PCR.

Our morphological and molecular identification results revealed that the isolated pathogenic fungus was *Fusarium oxysporum* f. sp. *lycopersici.* This confirmation was based on physical observation of white colour of the colony and a purplish-pink pigmentation produced by this isolate on the PDA medium. In addition to the physical observations, the morphological features showed typical spore topologies of FOL as described by Leslie and Summerell^44^, in their *Fusarium* spp. identification key, as reported by Mwangi et al.^46^ and Nagaraj et al.^47^. Furthermore, the identity of the FOL isolate was confirmed by molecular markers where the similarity of the pathogen from this study was linked to FOL isolates deposited in GenBank at 98.64 to 100%. Pathogenicity test was also conducted to assess if the identified fungus is avirulent or virulent in tomato cultivar Moneymaker which is known to be susceptible to fusarium wilt. The results showed stunting and yellowing of lower leaves in FOL-inoculated tomato seedlings; confirming that the isolated FOL was virulent in the host plant. These observations concurred with previous research reports on symptoms caused by FOL in infected tomato plants^16,48^.

The in vitro antagonistic effects assessment revealed that all the nine entomopathogenic and endophytic fungal isolates in this study were able to inhibit and/or suppress FOL mycelia growth. The entomopathogenic fungal isolates showed a lower FOL mycelia inhibition rate (less than 50%) compared to fungal endophytes whose inhibition rate was almost 100%. This could be due to slow growth rate of EPF compared to the pathogenic fungus, which could also be attributed to the inoculation technique. Ragavendran et al.^49^ demonstrated that, when *Beauveria* spp. and *Metarhizium* spp. are sub-cultured using streaking and spreading techniques, their growth and spread over the whole media increased. However, in this study, the EPF isolates were placed on the side of the plate using the agar block technique, following the protocol of dual culture assay. Furthermore, our observations also established that, in addition to the entomopathogenic effects of the tested EPF on insect pests^17^, they could also inhibit FOL pathogen at some extent. According to Shah et al.^50^, and Kumar et al.^51^, fungi in the order “Hypocreales” which encompass *Trichoderma* spp. and *Hypocrea lixii*, grow quickly and form concentric rings, giving them a strong competitive ability.

Although all the tested BCAs were able to inhibit FOL growth, three endophytic fungal isolates, *T. asperellum* M2RT4, *T. harzianum* KF2R41, and *H. lixii* F3ST1 outperformed all the other isolates with high inhibition rates. In this study, these isolates grew fast and hence overgrew, sporulated on the FOL colony, and impeded its growth. These results are in agreement with previous studies which reported the inhibitive and suppressive ability of *Hypocrea lixii* and *Trichoderma* species against various soil-borne pathogens^36,52–55^. In vitro studies conducted by^20,56^, *Trichoderma* spp. showed the potential of inhibiting *Sclerotium rolfsii* and *Fusarium oxysporum lycopersici* mycelial growth at almost 100% and 76.94% inhibition rates, respectively. In a similar study, four *Trichoderma* strains of *T. asperellum* TaspHu1, *T. harzianum* TharHu2, *T. hamatum* ThamHu3, and *T. atroviride* TatrHu4 were reported to cause more than 80% *Fusarium oxysporum* radial growth inhibition^36^. The ability of *H. lixii* and *Trichoderma* species to inhibit the growth of pathogens or plant pathogens has been attributed to their fast growth and competition for nutrients and space, as well as the production of enzymes responsible for degrading fungal cell walls such as α-1-3-glucanase, β-1-3-glucanase, and chitinase^13,26,52,57^.

The endophytic colonization assessment confirmed the presence of the artificially inoculated endophytes in all the tomato plants' parts. Endophytes are capable of forming symbiotic relationships with inoculated host plants by penetrating and colonizing the plant tissues, and this needs to be proven through their re-isolation from inoculated hosts^58^. In our study, endophytes *T. asperellum* M2RT4, *T. harzianum* KF2R41, and *H. lixii* F3ST1 colonized the roots, stem and leaves of tomato plants. However, the colonization differed across the isolates in various plant parts. For instance, *T. asperellum* M2RT4 had the highest colonization rate in inoculated tomato plants compared to *T. harzianum* KF2R41 and *H. lixii* F3ST1. These results were in agreement with previous studies that reported endophytic colonization of tomato and nightshade plants by various *Trichoderma* strains and *H. lixii* differed across the plant's parts^28,59^. Similarly, among tomato parts assessed, roots and leaves had the highest and lowest colonization rates, respectively. The low rate of colonization of leaves could likely be attributed to vertical transmission movement of endophytes in Phylum Ascomycota where the genus *Trichoderma* belongs^60^, and since our inoculation was done through seeds of the host plant. According to Robert and Brown^61^, endophytes develop within plant tissues from roots to leaves by ascending migration. This could explain the observed phenomena of low colonization rate in leaves compared to other plant parts. Furthermore, considering this study was conducted under controlled laboratory and greenhouse conditions where the seeds and soil were sterilized, to further confirm the colonization of the selected isolates under field conditions, we recommend field validation of the colonization of the selected isolates in future studies.

Beyond host colonization, the potential of *H. lixii* and *Trichoderma* spp. includes pest and disease control, increased plant biomass, as well as improved plant growth and yield^37,60,62–64^. In this study, endophytes *T. asperellum* M2RT4, *T. harzianum* KF2R41, and *H. lixii* F3ST1 were found to be highly effective in lessening incidence and severity of fusarium wilt disease caused by *Fusarium oxysporum lycopersici in-planta*, increasing dry weight of roots and shoots, as well as boosting growth and yield of colonized tomato plants. Results from this study demonstrated that tomato plants inoculated with the three potent endophytes were able to defend themselves against FOL as compared to control plants. This finding was also confirmed by the low incidence, severity and AUDPC of fusarium wilt in endophytically-colonized and FOL-infected tomato plants in comparison to FOL-infected tomato plants. For instance, fusarium wilt incidence was reduced by 96%, 88%, and 56% in FOL-infected tomato plants pre-treated with the three endophytes *T. asperellum* M2RT4, *H. lixii* F3ST1, and *T. harzianum* KF2R41, respectively (Table 1). Similar trends were observed in *T. asperellum* M2RT4, *H. lixii* F3ST1, and *T. harzianum* KF2R41, where disease severity reduced by 97.92%, 66.67%, and 44.44%, respectively (Table 2) through systemic resistance development of the host crop, and the effects of potential antifungal metabolites produced by the endophytes as well as natural mycoparasitism. These results are in line with previous studies' findings which reported reduced incidence and severity of *Fusarium* spp. in various host plants such as maize, melons and tomatoes^24,65,66^. Besides, the lower AUDPC were obtained in FOL infected tomato plants pre-treated with the selected endophytes compared to FOL-infected tomato plants untreated with endophytes (Fig S3). This observation indicated the effectiveness of endophytes against fusarium wilt. According to Sinno et al.^37^ and Baron and Rigobelo^60^, efficacy of *H. lixii* and *Trichoderma* spp. in disease suppression is related to indirect triggering of systemic defence in their host plants, as well as the production and release of secondary metabolites substances. This could be achieved through a priming mechanism which enables endophytically-colonized plants to respond quickly and defend themselves against pathogens^62,63,67^.

Significant increase in tomato plant growth parameters, as well as increased yield in FOL-infected tomato plants pre-treated with the three endophytes, concurs with previous studies that reported increased plant biomass and growth parameters in response to their treatment with *Trichoderma* spp.^28,36,62^. This growth stimulation might be attributed to endophytes producing phytohormones such as auxins and gibberellins which are important for the development of roots and shoots as well as fruit settings and maturity as reported by^36,60^. The three endophytes,* T*. *asperellum* M2RT4, *H. lixii* F3ST1, and *T. harzianum* KF2R41 were able to enhance plant height in FOL-infected tomato plants, with *H*. *lixii* F3ST1 recording the highest plant height. These findings concurred with the previously reported results by^28^. Our findings also agreed with previous studies that reported improved crop yield under *Trichoderma* spp. application regardless of the presence of the pathogen^62^.

Observing how potent endophyte *T. asperellum* M2RT4 is in colonizing tomato plants, lessening fusarium wilt incidence and severity while boosting tomato growth and yield, this study attempted to decipher the mechanism by which this isolate confers these benefits. To achieve this, the change in the expression levels of the TomPR1, TomPR2, TomloxC and SIWRKY in roots, stems and leaves of *T. asperellum* M2RT4-endophytically colonized, FOL-infected, *T. asperellum* M2RT4 endophytically colonized and FOL-infected tomato plants were assessed as compared to non-inoculated tomato plants. Our study revealed that the levels of expression of TomPR1, and TomPR2 genes were upregulated in the roots, stems and leaves of tomato plants after their exposure to the endophyte *T. asperellum* M2RT4 when compared to FOL-infected and un-exposed tomato plants. These findings agree with previous research studies that found enhanced expression of pathogenesis-related proteins PR-1 (TomPR1) and β-1,3-glucanases (TomPR2) in *Arabidopsis thaliana* and tomato plants treated with *Trichoderma* spp. in responses to bacterial and fungal pathogens such as *Fusarium oxysporum* and *Pythium aphanidermatum* invasions^52,68–70^. According to Win et al.^70^ and Soliman et al.^71^, TomPR1 and TomPR2 are responsible for defending host plant against fungal invasion by lysis of the pathogen cell wall thereby suppressing pathogenic fungal growth. In this study, we also observed a significant increase in expression level of TomLoxC gene in the roots, stems and leaves of *T. asperellum* M2RT4 endophytically-colonized tomato plants, as well as in *T. asperellum* M2RT4 endophytically-colonized and FOL-infected tomato plants compared to FOL-infected and un-inoculated tomato plants. Although previous studies only identified TomloxC expression in tomato fruits, our findings revealed that TomloxC activity began during the crop’s vegetative phase^30,72,73^. Furthermore, our results also showed that *T. asperellum* M2RT4 induced significant upregulation of the SIWRKY4 gene in both roots, stems and leaves of colonized tomato plants and in FOL-infected plants.

## Conclusion

Our findings proved that the two endophytes *H*. *lixii* F3ST1 and *T. asperellum* M2RT4 could successfully control *Fusarium oxysporum lycopersici* while boosting the yield of tomato crop. Thus, these endophytes could be formulated into biopesticides to sustainably control FOL pathogen and consequently reduce fusarium wilt disease incidence and severity in tomato cropping systems. Furthermore, the endophyte *T. asperellum* M2RT4 triggered the expression of Pathogenesis Related Protein-1 (TomPR1), β-1,3-glucanases (TomPR2), TomloxC, and SIWRKY4 genes, whose main function is to protect host plants against fungal invasion for improved yield quantity and quality^31,70,71^. The upregulation of the studied genes was negatively correlated with reduced incidence and severity of fusarium wilt in *T. asperellum* M2RT4 endophytically-colonized tomato plants. Therefore, the higher expression of the assessed tomato defence genes and reduced severity of FOL disease in *T. asperellum* M2RT4 colonized than in un-inoculated tomato plants, indicate that the endophyte *T. asperellum* M2RT4 is able to confer systemic resistance in tomato plants against *Fusarium oxysporum lycopersici.* Further studies should also be warranted in exploring the host plant detection and reaction mechanisms to the beneficial fungi based on microbe-associated molecular patterns as previously demonstrated by Bittel and Robatzek^74^ and Boller and Felix^75^.

## Material and methods

### Study site

In vitro and greenhouse experiments were conducted at the International Centre of Insect Physiology and Ecology (*icipe*), Duduville campus, Nairobi, Kenya (S 01° 13′ 14.6″; E 036° 53′ 44.5″), 1612 m above sea level. For the bioprospecting study of the plant pathogen, roots and stem samples with brown discolouration of the vessels in their cross-sections from tomato plants with yellowing of the lower leaves, typically confined to one side of the host plant were collected. The samples were collected in Mwea—Kirinyaga county, Kenya (S-0.628516 E37.374851, 1163 m) for *Fusarium oxysporum lycopersici* (FOL) isolation.

### Test pathogen isolation and identification

The field-collected samples were cut into small pieces of 1 cm for roots and stems targeting disease leading edge which is mainly characterized by high pathogen activity^76^. Samples were surface sterilized by submerging them in 70% ethanol for 1 min and then 1.5% sodium hypochlorite for 2 min. They were then rinsed three times for one minute with distilled water to wash out the sodium hypochlorite and blot-dried on a paper towel under a laminar flow hood. Afterwards, using sterile forceps, each piece of the blot-dried sample was placed into Petri dishes containing Potato Dextrose Agar (PDA) medium supplemented with 0.1% chloramphenicol antibiotic. The Petri dishes were then sealed with parafilm and incubated at 25 ± 2 °C for 7 days. Fungal colonization was assessed in each isolated plant tissue and actively growing fungi were isolated and sub-cultured onto new PDA plates for purification. Several subcultures were conducted to acquire pure fungal isolates before their identification. The pure cultures were obtained by transferring the mycelia to Petri dishes containing PDA medium and incubated as mentioned above.

To confirm whether the isolated fungus was FOL, macroscopic, microscopic, and molecular identification were conducted. Macroscopically, FOL was identified through physical observation of the colony morphology and pigmentation of the 7-day pure culture plates of the isolates, and by morphological key described by Leslie and Summerell^44^, as reference. The same morphological key was used to identify the isolates microscopically based on the shape and size of the spores displayed with the ZEISS Primo Star microscope at 40× magnification. For molecular identification, total DNA was extracted from pure cultures of the fungi using the Isolate II Plant DNA Extraction Kit (Bioline, London, UK) as per the manufacturer’s instructions. The extracted DNA quality and quantity were assessed using a Nanodrop 2000/2000c spectrophotometer (Thermo Fischer Scientific, Wilmington, USA), and stored at − 20 °C, until further processing. The intergenic transcribed spacer region ITS 5 and ITS 4 primers, (5ʹ-TCCTCCGCTTATTGATATGC-3ʹ) and (3ʹ-GGAAGTAAAAGTCGTAACAAGG-5ʹ), elongation factor 1-α EF1-983 (5ʹ-GCY CCY GGH CAY CGT GAY TTY AT-3ʹ) and EF1-2218 (3ʹ-AT GAC ACC RAC RGC RAC RGT YTG-5ʹ), largest subunit of RNA polymerase RPB1-A (5ʹ-GARTGYCCDGGDCAYTTYGG-3ʹ) and RPB1-C (3ʹ-CCNGCDATNTCRTTRTCCATRTA-5ʹ) were used for amplification of the target regions. The samples were sent to Microgen for sequencing and the obtained sequences were edited and assembled using BioEdit Sequence Alignment Editor Version 7.2.5^77^. The sequences were then matched with those found in GenBank of the National Center for Biotechnology Information (NCBI) through the Basic Local Alignment Search Tool (BLAST)^78,79^. The sample with a similarity index of 99–100% was considered as the positive FOL.

### Preparation of biocontrol fungi

Both the endophytic and entomopathogenic fungal isolates were obtained from *icipe*’s Arthropod Pathology Unit (APU) Germplasm for the bioassays. The endophytic fungal isolates *Hypocrea lixii* F3ST1, *Trichoderma asperellum* M2RT4, *T. atroviride* ICIPE 710, and *T. harzianum* KF2R41 which were already reported as endophytes in tomato, nightshade and beans were used^17,28,55^. The entomopathogenic fungal isolates that were used were: *Beauveria bassiana* isolates (ICIPE 706 and ICIPE 273) and *Metarhizium anisopliae* isolates (ICIPE 18, ICIPE 20 and ICIPE 665) that have been previously reported to be effective against the tomato leafminer, *Phthorimaea absoluta* formerly known as *Tuta absoluta*^59,80,81^. The endophytic and EP fungal isolates have been selected based on their ability to colonize all tomato plant parts, control plant pests (*T. absoluta* (Meyrick), whiteflies, (*Trialeurodes vaporariorum)*, and enhance crop performance, as reported by^28,59,81^.

These above isolates were subcultured on a PDA medium and incubated at 25 ± 2 °C for further use, except for *Metarhizium anisopliae* isolates which were maintained on Sabouraud dextrose agar (SDA) medium in complete darkness. The inoculum preparations for all the fungal isolates were done by harvesting conidia by scraping the surface of 2-week-old pure fungal cultures that were sporulating using a sterile spatula. The harvested conidia were mixed in 10 mL of sterile distilled water containing 0.05% (w/v) Triton X-100 (EMD Millipore Corporation, USA) in a universal bottle and vortexed for 5 min at about 700 rpm to produce homogenous conidial suspensions. Conidial counts were performed with an improved Neubauer Hemocytometer^82^. The inocula were adjusted to a concentration of 1 × 10^8^ conidia mL^−1^.

Before any bioassay, the spore viability test was performed by spreading 0.1 mL of 3 × 10^6^ conidial mL^−1^ onto 9 cm Petri dishes containing SDA or PDA. Plates were sealed with parafilm and incubated at 25 ± 2 °C in complete darkness for 15–18 h, depending on the fungal isolate as some germinate faster than others^59^. The isolates were set in quadruplets and arranged in a completely randomized design (CRD). After 15–18 h, the plates were unsealed, fixed with a drop of fixative lactophenol cotton blue, and randomly covered with four sterile microscope coverslips (2 × 2 cm)^59^. Percentage conidial germination was determined from 100 randomly selected conidia on the surface covered by each coverslip under a light microscope (40×)^82^. A conidium was considered to have germinated when the length of the germ tube was at least twice its diameter^83^.

### In vitro assessment of fungal biocontrol agents against *Fusarium oxysporum lycopersici*

Using the dual culture assay, both EPF and endophytic fungal isolates were screened for their antagonistic activity against the FOL pathogen. Zero-point five centimeter (0.5 cm) of the mycelial disc from a 5-day-old culture of FOL was placed at 1 cm from the end of the 9 cm Petri dish with PDA. A similar disc of EPF or endophytic fungal isolates was placed opposite the mycelial disc of FOL. Plates solely inoculated with FOL served as control treatment. Each treatment was replicated four times, sealed and incubated in an inverted position at 25 ± 2 °C. The radius of mycelial growth of FOL, in both dual culture and in control, was measured with a ruler every 24 h until a pathogen completely covered the surface of the control plate. Percentage inhibition of radial growth was obtained using the formula $\left[\left(\text{R}1-\text{R}2\right)\times 100/\text{R}1\right]$ as described by Myrchiang and Devi^84^, where R1 is the radius of radial growth of the pathogen towards the opposite side of the control plate, and R2 is the radius of radial growth of the pathogen towards the antagonist in dual culture.

To further screen for the antagonistic ability of the beneficial fungal isolates against FOL, the co-culture assay was also performed following the modified protocol described by Karuppiah et al.^85^. Zero-point one milliliter (0.1 mL) of 1 × 10^8^ conidia mL^−1^ of FOL and fungal biocontrol agent (BCA) were spread together on a Petri dish with PDA. Single cultures of FOL served as control treatment. Plates were sealed with parafilm and incubated at 25 ± 2 °C for 14 days. Each Petri dish from the treatments and control was replicated four times. The experiment was set in a completely randomized design. Appearance, dominance of mycelia, and pigmentation produced by FOL/BCAs in single-cultured and dual-cultured plates were recorded by visual observation.

### Suspension preparation and inoculation of the tomato seeds with the selected potent biocontrol agents

The tomato cultivar “Money-maker” seeds (SimLaw Seeds Ltd.) were bought in Agroduka, Nairobi, Kenya. The seeds were surface sterilized by soaking them in 70% ethanol for 2 min, followed by a 1.5% solution of sodium hypochlorite for 3 min with continuous shaking and rinsed three times in sterile distilled water for 1 min^28^. Seeds were dried at room temperature on a sterile paper towel for 3 h in a laminar flow hood^86^. The effectiveness of the surface sterilization procedure was done by plating the last rinse water using a sterile loop on a Petri dish containing PDA medium and also imprinting of surface sterilized seeds onto PDA (tissue imprint) supplemented with 100 mg/L Streptomycin and plates were incubated at 25 °C for 14 days^19,24,55,86,87^. Then, the absence of fungal growth after incubation indicated the successfulness of the sterilization procedure^28^. The seed inoculation was done by placing the surface-sterilized tomato seeds into the suspension of the potent endophytic fungal isolates at a concentration of 1 × 10^8^ conidia mL^−1^ for 18 h before sowing them^28^. One portion of the seeds was left un-inoculated with antagonistic fungal isolates to serve as a control.

### Endophytic colonization and pathogenicity assessment

The endophytes *T. asperellum* M2RT4, *H. lixii* F3ST1 and *T. harzianum* KF2R4 which exhibited more than 80% FOL mycelial growth inhibition during in vitro study, were selected for *in-planta* experiments. Both the inoculated and un-inoculated seeds were sown in pots of 8 cm diameter and 7 cm height filled with 1 kg of the sterile mixture ratio of 5:1 field soil and compost which were autoclaved at 121 °C for 2 h and left to cool for 72 h before their use. Five seeds were sown per pot and thinned to two after 2 weeks. The seedlings were maintained at room temperature in a greenhouse under natural light conditions with no additional fertilizer^28,59,80^. The seedlings were watered regularly once in 2 days, using sterile distilled water to make sure that nothing else would affect the experiment, except the planned treatments.

To confirm the presence of endophytes in tomato tissues, five randomly selected 3-week-old tomato seedlings were uprooted from their pots and washed with tap water to remove the soil. Plants were divided into three parts (root, stem, and leaves), cut into 1 cm root and stem pieces and 1 cm^2^ leaf pieces, and surface sterilized in a laminar flow hood as described by Paradza et al.^28^. Five pieces were placed on a 9 cm diameter Petri dish containing PDA enriched with antibiotic (25 μg/ml chloramphenicol) and incubated at 25 ± 2 °C for 2 to 3 weeks^28^. The number of plant pieces showing fungal outgrowth divided by the total number of plant pieces plated was used to compute the proportion of the plant parts colonized by the inoculated fungal isolate for each treatment^28^. The assessment was based on the morphological characteristics of the inoculated fungus that colonized the incubated plant part, and only the colonization by the inoculated fungus was recorded as positive^28^.

To confirm the virulence of isolated FOL, each pot with 4-week-old tomato seedlings was soil-drenched with 10 mL of FOL inoculum at a concentration of 1 × 10^8^ conidia mL^−1^. The control treatment pots were soil-drenched with a solution of Triton X-100 and sterile distilled water without FOL. Pots were kept in the greenhouse for further inspection of the occurrence and severity of fusarium wilt disease symptoms as described by Seo and Kim^88^ and Andrade-Hoyos et al.^55^, respectively. Afterwards, FOL was re-isolated from diseased tissues to complete Koch’s postulates^1^.

### *In* *planta* evaluation of the selected potent antagonists against Fusarium wilt

Both inoculated and un-inoculated tomato seeds were sown in pots of 16.5 cm base diameter, 18 cm tall, and 22.5 cm top diameter (5-L pot) filled with a sterile mixture of topsoil and well-decomposed goat manure at a ratio of 5:1. The planting media were previously soil-drenched with 10 mL of FOL inoculum at a concentration of 1 × 10^8^ conidia mL^−1^ on the same day. The experiment consisted of eight treatments with endophytically colonized tomato seedlings and FOL inoculum as follows: (i) *T. asperellum* M2RT4 and FOL; (ii) *T. harzianum* KF2R41 and FOL; (iii) *H. lixii* F3ST1 and FOL; (iv) *T. asperellum* M2RT4 alone; (v) *T. harzianum* KF2R41 alone; (vi) *H. lixii* F3ST1 alone; (vii) endophyte-free seedlings (negative controls) and with FOL; and (viii) endophyte-free seedlings alone without FOL (positive control). Each treatment consisted of a 5 L pot with two seedlings in each pot. A completely randomized design was used with three replications over time. The seedlings were kept inside a greenhouse and maintained as described above.

### Fusarium wilt disease assessment

Fusarium wilt disease incidence and severity data were collected at 15-, 30-, 45-, and 60-days post inoculation (dpi) on FOL-inoculated tomato plant in reference to leaf wilting and vascular discolouration. The incidence was determined using the following formula:

$\text{Disease incidence }\left(\text{\%}\right)\text{ = }\left(\text{Number of infected plants}/\text{Total number of inoculated sampled plants}\right)\times {{100}}$ adopted from^55^, while the severity was assessed using a 0–4 ranking scale, as described by Amini^89^ and Horinouchi et al.^90^.

The Area Under Disease Progress Curve (AUPDC) was determined as explained by Batista^91^ using a formula:

$$\text{AUPDC}=\sum \limits_{i=1}^{n}\left[\left(\frac{{Y}_{i+1} +Yi}{2}\right)\left({T}_{i+1} -{T}_{i}\right)\right]$$

where: Y_i_ = Severity of fusarium wilt at the ith observation, T_i =_ time (days after inoculation) at the ith observation and n = total number of evaluations.

### Plant growth and yield parameters

Tomato growth parameters such as plant height, number of completely developed leaves, leaf width, and leaf length were assessed as defined by^92^. The dry weights of roots and shoots were obtained by placing them in an oven at 60 °C for 48 h and then weighed using Endeavour electronic weighing balance with a precision of ± 0.001 g^93^.

Three yield parameters such as number of fruits, fruit weight, and yield were collected in the manner outlined by^94^, where the total number of fruits per plant and the individual fruit weight were obtained by counting and weighing each marketable fruit. The yield (kg plant^−1^) was obtained by totalling the weight of all fruits harvested from each plant. The yield estimation in tons per ha was calculated using the below formula described by Ali et al.^95^:

$$\text{Yield }(\text{T }{\text{ha}}^{-1})= \frac{\text{Yield per pot }\left(\text{kg}\right) \times {10,000}}{\text{Area occupied by pot }\left({\text{m}}^{2}\right)\times {1,000}}$$

### Tomato defence gene expression

Based on the *in planta* experiment results above, the endophyte *T. asperellum* M2RT4, which displayed a high colonization rate, with complete suppression/inhibition of fusarium wilt pathogen, disease incidence and severity was selected and assessed for its ability to trigger the defence gene in the endophytically-colonized tomato crop. Five-liter pots were filled with a sterile mixture of topsoil and goat manure at a 5:1 ratio. The pots were soil-drenched with 10 mL of FOL inoculum at a concentration of 1 × 10^8^ conidia mL^−1^. Afterwards, *Trichoderma asperellum* M2RT4 inoculated tomato seeds were sown into the planting medium on the same day. The experiment consisted of four treatments: (i) FOL-infected tomato plants, (ii) *T. asperellum*-endophytically colonized tomato plants, (iii) *T. asperellum* M2RT4 endophytically-colonized and FOL-infected tomato plants, and (iv) un-inoculated tomato plants (control). Each treatment had six replicates and was organized in a completely randomized design.

One (1) g of roots, stems, and leaves of each treatment sample were cut and placed into 2 mL Eppendorf tubes and stored at − 80 °C for RNA extraction. RNA extraction was done using the ISOLATE II RNA Mini Kit, (Bioline, London, UK) following the manufacturer’s guidelines. The RNA samples were reverse-transcribed into cDNA using SensiFAST cDNA Synthesis Kit (Meridian Bioscience, London, UK) following the manufacturer’s guidelines. The target primers were designed using primer 3.0 software, available at NCBI (https://www.ncbi.nlm.nih.gov/tools/primer-blast/) (Table 7). The qPCR reaction was performed using QuantStudio 5 Real-Time PCR system (Thermo Fisher, Scientific, Waltham, USA). PCR mixture constituted of PCR water (1.5 μL), SensiFAST SYBR® Hi-ROX Kit (5 μL), Forward primer (0.5 μL) and Reverse primer (0.5 μL) and RNA template (2.5 μL). Each qPCR reaction consisted of both positive (known sample) and negative (non-template control) samples. GAPDH1 was used as a housekeeping gene to normalize gene expression. Relative expression of tomato defence-related genes was analyzed using the delta-delta Ct method (2^−ΔΔCt^)^45^.

**Table 7:** Primers for defence-related genes for tomato plants and reference genes.

Primer name	Target gene	Sequence (5ʹ–3ʹ)	Source/accession no
*TomLoxC*-Fwd	Tomato lipoxygenase	TCCGGCAACACCGTTTACTC	U37839
*TomLoxC*-Rev	Tomato lipoxygenase	GTCAATGGCCGGAAAATGTG	
TomPR2-Fwd	β-1,3, glucanase	AAGTATATAGCTGTTGGTAATGAA	NM001247229
TomPR2-Rev	β-1,3, glucanase	ATTCTCATCAAACATGGCGAA	
SIWRKY4-Fwd	WRKY gene family	CGTTGCACATACCCTGGAGT	XP_004235494.1
SIWRKY4-Rev	WRKY gene family	GGCCTCCAAGTTGCAATCTC	
TomPR1-Fwd	Pathogenesis-related protein-1	GGATCGGACAACGTCCTTAC	Y08804
TomPR1-Rev	Pathogenesis-related protein-1	GCAACATCAAAAGGGAAATAAT	
GAPDH-Fwd	Reference gene	GGATTACAAGGAAAAGTTCCACGA	NC_015440.3
GAPDH-Rev	Reference gene	GTGCTCACCAAAACTCAA	

### Data analyses

Before data analysis, the normality test was conducted to confirm if the data were normally distributed using the Shapiro–Wilk test^96^. Data on colonization rate, % inhibition of radial growth, and incidence and severity of fusarium wilt disease were analyzed using the generalized linear model (GLM) package of R statistical software^97^, assuming a binomial distribution with the log link function. The number of leaves and fruits per plant data were analyzed using GLM quasi-Poisson. Data on leaf width and length, plant height, fresh and dry weight of roots and shoots were analyzed using analysis of variance. The relative expression of tomato defence genes was analyzed using the delta-delta Ct method (2^−ΔΔCt^)^45^. All statistical analyses were done using R version 4.3.1^97^. The treatment means were separated using the Student Newman Keuls (SNK) test. A p-value of less than 0.05 was considered for statistical significance.

### Ethics approval

The experimental research and field studies on plants, including the collection of plant material, complied with relevant institutional, national, and international guidelines and legislation. The appropriate permissions and/or licenses for collection of plant or seed specimens were obtained for the study as approved by the National Commission of Science, Technology and Innovations, Kenya (License No: NACOSTI/P/23/23608). This article does not contain any studies with human participants performed by any of the authors.

### Supplementary Information

Supplementary Figures.

## Data Availability

All relevant data are within the paper and supplementary materials.
