# Supplementary material for: Biostimulant and antagonistic potential of endophytic fungi against fusarium wilt pathogen of tomato Fusarium oxysporum f. sp. lycopersici
Source: Sci Rep. 2024 Jul 4;14:15365. doi: 10.1038/s41598-024-66101-1 (PMC11224277; doi:10.1038/s41598-024-66101-1)
Supplement: Supplementary file 1 — Supplementary Figures. [file 41598_2024_66101_MOESM1_ESM.docx]

**Biostimulant and antagonistic potential of entomopathogenic and endophytic fungi against fusarium wilt pathogen of tomato Fusarium oxysporum f. sp. lycopersici**

Marie Cecile Muhorakeye^1,2,3^, Everlyne Samita Namikoye^2^, Fathiya M. Khamis^1^, Waceke Wanjohi^2^, Komivi S. Akutse^1,4*^

^1^International Centre of Insect Physiology and Ecology (*icipe*), P.O. Box 30772-00100, Nairobi, Kenya.

^2^Kenyatta University, Department of Agricultural Science and Technology, P.O. Box 43844-00100, Nairobi, Kenya.

^3^Rwanda Polytechnic, Integrated Polytechnic Regional College (IPRC) Musanze, P.O. Box 226 Musanze, Rwanda

^4^Unit of Environmental Sciences and Management, North-West University, Private Bag X6001, Potchefstroom 2520, South Africa.

^*^Corresponding author: [kakutse@icipe.org](mailto:kakutse@icipe.org)

**Supplementary Figures**


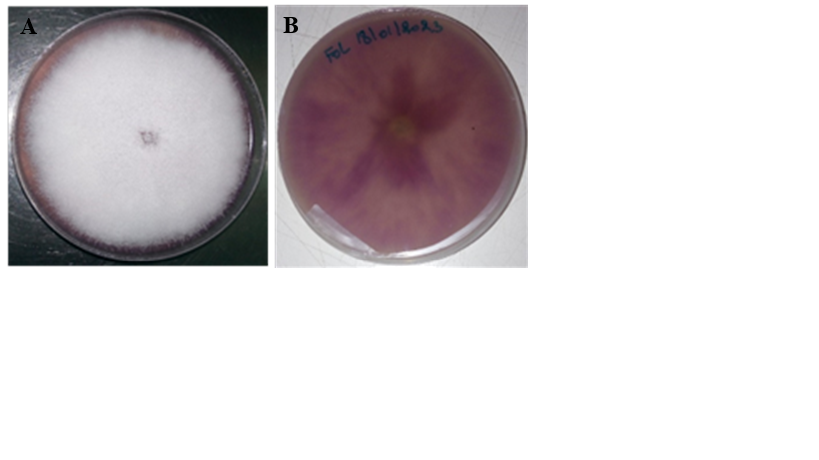


**Figure S1.** Morphological identification of *Fusarium oxysporum lycopersici* (FOL). Images **A** and **B** show the upper and basal surfaces of the FOL colony on the PDA medium, respectively.

| 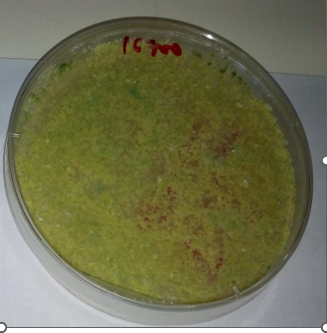 | 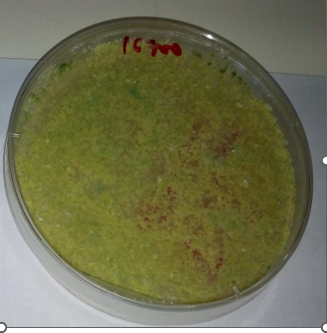 | 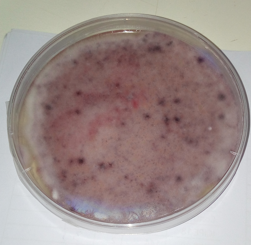 | 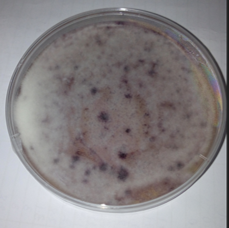 |
| --- | --- | --- | --- |
| Co-culture of *T. asperellum* M2RT4 and FOL at 7^th^ day of co-culture assay | Co-culture of *T. asperellum* M2RT4 and FOL at 14^th^ day of co-culture assay | Co-culture of FOL and *B. bassiana* ICIPE 273 at 7^th^ day of co-culture assay | Co-culture of FOL and *B. bassiana* ICIPE 273 at 14^th^ day of co-culture assay |
| 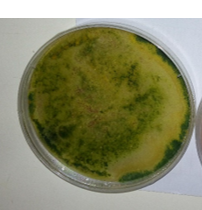 | 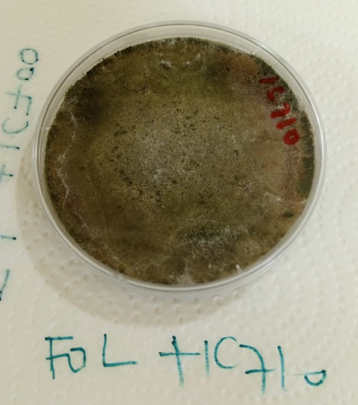 | 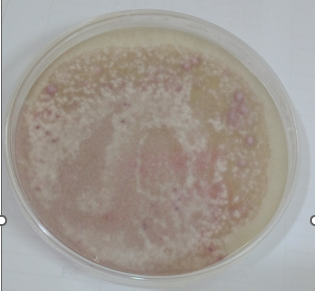 | 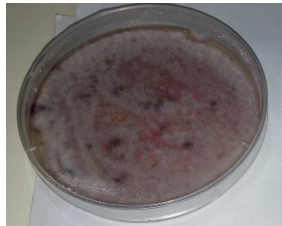 |
| Co-culture of *T. atroviride* ICIPE710 and FOL at 7^th^ day of co-culture assay | Co-culture of *T. harzianum* KF2R41 and FOL at 14^th^ day of co-culture assay | Co-culture of FOL and *M. anisopliae* ICIPE 20 at 7^th^ day of co-culture assay | Co-culture of FOL and *M. anisopliae* ICIPE 20  at 14^th^ day of co-culture assay |
| 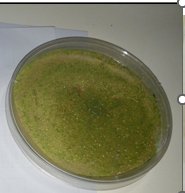 | 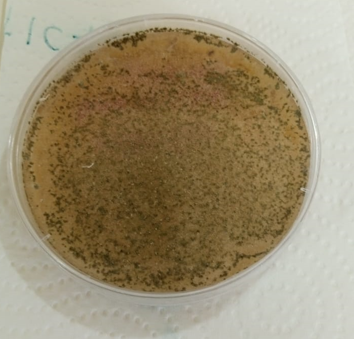 | 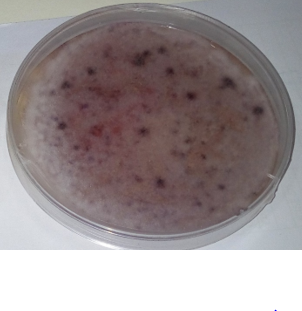 | 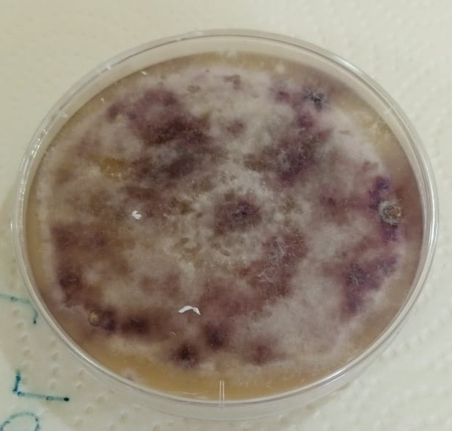 |
| Co-culture of *T. harzianum* KF2R41 and FOL at 7^th^ day of co-culture assay | Co-culture of *T. harzianum* KF2R41 and FOL at 14^th^ day of co-culture assay | Sole culture of FOL (control) at 7^th^ day of co-culture assay | Sole culture of FOL (control) at 14^th^ day of co-culture assay |

**Figure S2.** Morphological observation of dominance in mycelia and pigment produced by the selected fungal endophytes and entomopathogens on the 7^th^ day and 14^th^ day of the co-culture

**Figure S3**. Area Under Disease Progress Curve (AUDPC) for the *Fusarium oxysporum lycopersici* infected tomato plants pre-treated with the selected endophytes and the control treatment (CT).
